# First-World Care at Third-World Rates: Pakistan, an Attractive Destination for Bariatric Tourism

**DOI:** 10.7759/cureus.48572

**Published:** 2023-11-09

**Authors:** Umar Bashir, Ghulam Siddiq, Nitasha Saleem, Humza Farooq, Muhammad Awais, Muhammad Ussama, Hania Iqbal, Hassan Shabbir, Talha Rafiq, Mustafa Banoori

**Affiliations:** 1 Surgery, Shifa International Hospital Islamabad, Islamabad, PAK; 2 Surgery, Hamad General Hospital, Doha, QAT; 3 General Surgery, Shifa International Hospital Islamabad, Islamabad, PAK; 4 Surgery, Sir Gangaram Hospital, Lahore, PAK; 5 Surgery, Combined Military Hospital, Multan, PAK; 6 Surgery, Shifa Tameer-E-Millat University Shifa College of Medicine, Islamabad, PAK

**Keywords:** sleeve gastrectomy, gastric bypass, weight loss surgery, bariatric tourism, obesity, bariatric surgery, medical tourism

## Abstract

Introduction

Obesity, a complex and multifactorial disease, is defined by a body mass index (BMI) greater than 30 kg/m². When the BMI exceeds 40 kg/m², it is classified as morbid obesity. This condition leads to excessive fat accumulation, which impairs normal body function and metabolism.

For individuals grappling with morbid obesity and those who have faced significant hurdles in their quest for substantial weight loss, bariatric surgery emerges as a vital option.

Purpose

The study aims to explore the dynamics of bariatric surgical tourism in Pakistan, shedding light on factors influencing the choice of Pakistan as a destination for bariatric tourism.

Materials and methods

A retrospective cross-sectional study design was adopted. Data were gathered from the medical records database, including all patients who had undergone bariatric surgery from 2018 until 2022. The data collection process involved comprehensive patient outreach, where investigators conducted phone interviews and collected patient satisfaction assessments. During these phone interviews, valuable information was gathered by posing questions. These inquiries encompassed various aspects, including the patient's overall satisfaction with the surgical experience, their countries of origin, the specific bariatric procedures they underwent, the motivating factors behind their decision to travel abroad for surgery, their postoperative follow-up routines, and any complications they may have encountered.

Results

One hundred and nine patients traveled to Pakistan for bariatric surgery from 2018 to 2022. Out of 109 patients, 78 responded to the questionnaire by phone or email. The proforma was filled by 41 (52.5%) males and 37 (47.5%) females. Forty-seven (60.2%) of these patients underwent Roux-en-Y gastric bypass and 31 (39.8%) patients underwent sleeve gastrectomy. Out of 78 bariatric patients, 72 (92.3%) were satisfied with their surgery, five patients (6.4%) were neutral in their response and one patient (1.3%) was dissatisfied with the surgery. Most of the patients (26, 33.3%) declared money as the main driving force for traveling, with long waiting times being the close second reason (19, 24.36%) patients.

Conclusion

At least 2% of worldwide bariatric procedures are provided for medical tourists. Countries such as Mexico, India, Lebanon, and Romania dominate as providers for patients mainly from the USA, UK, and Germany. The lack of affordable bariatric healthcare and long waiting lists are some of the reasons for patients choosing bariatric tourism. The 92.3% satisfaction rate of patients with the surgery and its outcomes is a significant finding, as it suggests that bariatric surgery services provided in Pakistan are meeting or exceeding the expectations of international patients. The exceptionally high level of patient satisfaction speaks to the quality of care provided by the medical institutions in Pakistan. The data and analysis presented in this study shed light on the motivations and experiences of international patients traveling to Pakistan for bariatric surgery. These insights are invaluable for healthcare providers, policymakers, and the medical tourism industry as they seek to enhance the accessibility, affordability, and quality of healthcare services for domestic and international patients.

## Introduction

Obesity, a complex and multifactorial disease, is defined by a body mass index (BMI) of greater than 30 kg/m². When the BMI exceeds 40 kg/m², it is classified as morbid obesity [1]. This condition leads to excessive fat accumulation, which impairs normal body function and metabolism. For individuals grappling with morbid obesity and those who have faced significant hurdles in their quest for substantial weight loss, bariatric surgery emerges as a vital option

It is the second leading cause of preventable death following smoking in the United States [2]. Obesity requires multi-pronged and lifelong treatment. The myriad treatment options range from diet restrictions, diet therapy, physical exercise, anti-obesity drugs, and bariatric surgery [3-5].

Bariatric surgery emerges as a vital option for individuals grappling with morbid obesity, those who have faced significant hurdles in their quest for substantial weight loss. This option becomes particularly pertinent when conventional weight loss methods, such as diet and exercise, prove less effective and when the burdens of obesity begin to take a toll on one's mobility, cardiovascular well-being, and overall mental and physical health [6].

Bariatric surgery, encompassing procedures such as gastric bypass and sleeve gastrectomy, has emerged as a pivotal intervention for combating obesity-related health issues globally [7]. The demand for bariatric surgeries continues to rise, driven by the escalating prevalence of obesity and its associated comorbidities such as diabetes, cardiovascular diseases, and hypertension [8]. These procedures go beyond extending patient lifespans; they also address and resolve complications related to obesity, such as diabetes, hypertension, and hyperlipidemia, significantly improving overall health [9,10].

Medical tourism has gained substantial attention in recent years, driven by the increasing globalization of healthcare services and the quest for cost-effective, high-quality medical treatments. A noteworthy facet of this phenomenon is the surge in bariatric surgery tourism, where individuals travel abroad to undergo weight loss procedures. This research delves into the burgeoning field of bariatric surgery tourism within Pakistan, which has witnessed a noteworthy influx of international patients seeking weight loss solutions.

Simultaneously, medical tourism, characterized by the deliberate cross-border movement of patients for medical treatment, has substantially expanded. With its competitive pricing, highly trained medical professionals, and state-of-the-art healthcare facilities, Pakistan has emerged as a burgeoning destination for bariatric surgery tourists.

However, despite the growing popularity of bariatric surgery tourism in Pakistan, comprehensive research on this topic remains limited. This study aims to bridge this gap by exploring the dynamics of bariatric surgery tourism in Pakistan, shedding light on factors influencing the choice of Pakistan as a destination, patient satisfaction levels, the economic impact on the healthcare system, and the implications for healthcare providers and policymakers.

In a global context where healthcare access and affordability are paramount concerns, understanding the landscape of bariatric surgery tourism in Pakistan holds significance for the country and the broader discourse on medical tourism and its implications on global healthcare systems.

## Materials and methods

It is a retrospective, unicentric, cross-sectional study wherein data was gathered from the medical records database after the approval of the institutional review board. All electronic data of patients who underwent bariatric surgery from January 2018 up till January 2022 were gathered.

Inclusion criteria specifically targeted individuals who had journeyed to Pakistan with the sole purpose of receiving bariatric surgery from January 2018 to January 2022 irrespective of gender and age. Meanwhile, exclusion criteria involved patients who were residents of Pakistan or had resided in the country for a duration exceeding three months before their surgical procedures and patients who had previous bariatric surgery and had presented for revision bariatric surgeries irrespective of where they had traveled from.

The data collection process involved comprehensive patient outreach, where investigators conducted phone interviews with all patients who underwent bariatric surgery from January 2018 to January 2022. Some patients who volunteered to fill out the questionnaire themselves were emailed the questionnaire, which they filled out and sent back to us.

During the specified time period, a total of 109 bariatric patients who had traveled from other countries underwent surgical procedures. The contact information, including phone numbers and email addresses, for all 109 patients was obtained from our database.

All the patients were contacted via phone calls, during which we sought and received their verbal informed consent to participate in the study. Out of the 109 patients, 78 patients willingly agreed to take part in the study.

In the course of these phone interviews and email correspondences, patients were asked a set of questions. These encompassed a wide range of inquiries, such as the patient's demographics, their countries of origin, the specific bariatric procedures they had undergone, their overall satisfaction with the surgical experience, the driving factors behind their decision to seek surgery abroad, their postoperative follow-up routines, and any complications they may have encountered. To measure patient satisfaction, we employed a five-point Likert satisfaction scale. This enabled us to collect insights from willing participants, shedding light on various aspects of their experiences.

All statistical analysis was performed using Statistical Package for the Social Sciences version 21.0 (SPSS; IBM Inc., Armonk, New York). All tables and figures were made using Microsoft Excel 2016 (Microsoft, Redmond, Washington).

## Results

A total of 78 out of 109 international patients took part in the study; these were patients who traveled to Pakistan for bariatric surgery between 2018 and 2022. The gender distribution in the respondent group was relatively even, with 52.5% males and 47.5% females (Table 1).

**Table 1:** Female/male ratio

	Numbers	Percentage
Male	41	52.5%
Female	37	47.5%

The most commonly performed bariatric procedures were Roux-en-Y gastric bypass (60.2%) and sleeve gastrectomy (39.8%) (Table 2).

**Table 2:** Number of Roux-en-Y vs sleeve gastrectomy patients

	Number	Percentage
Roux-en-Y gastric Bypass	47	60.2%
Sleeve Gastrectomy	31	39.8%

**Figure 1:**
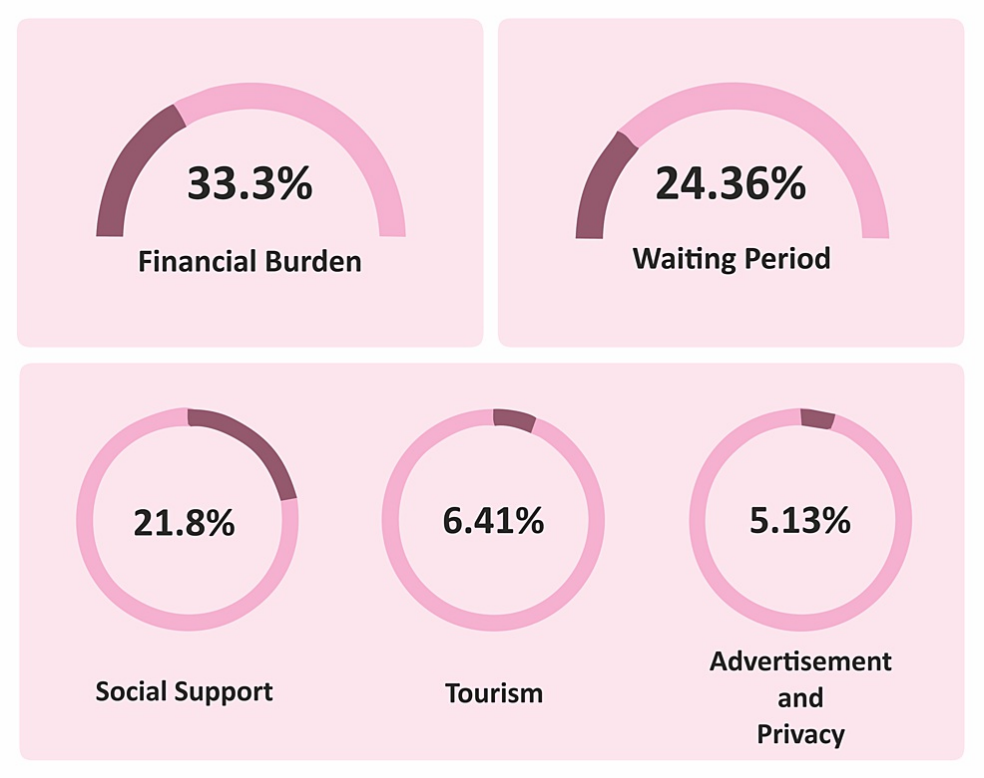
Motivation factors

Results from the study showed a remarkably high satisfaction rate among the surveyed patients. An overwhelming majority (92.3%) expressed satisfaction with their surgical procedures (Table 3, Figure 1).

**Table 3:** Percentage of patient satisfaction

	Number	Percentage
Satisfaction	72	92.3%
Neutral	5	6.4%
Dissatisfaction	1	1.3%

The motivations driving patients to choose Pakistan for bariatric surgery; the most prominent factors motivating patients were economics and a long waiting list. Thirty-three point three percent (33.3%) cited cost savings as the primary driving force and 24.36% mentioned long waiting time as a driving force (Table 4).

**Table 4:** Motivation factors

Factors	Numbers	Percentage
Financial Burden	26	33.3%
Waiting period	19	24.36%
Social support	17	21.8%
Tourism	5	6.41%
Advertisement	4	5.13%
Privacy	4	5.13%
Unavailability in the home country	-	0%
Better medical services	-	0%

Our data reveal a diverse range of countries of origin for these international patients as well. The largest group came from Qatar (22 patients), followed by Afghanistan (15 patients), the United Kingdom (13 patients), the United Arab Emirates (nine patients), Saudi Arabia (seven patients), the United States of America (five patients), Canada (three patients), and Australia (one patient).

## Discussion

The burgeoning phenomenon of bariatric tourism is gaining prominence among individuals seeking cost-effective and high-quality healthcare options. This emerging trend offers several notable advantages, including cost reduction, decreased waiting times, access to cutting-edge medical technologies and expertise, and the prospect of combining medical procedures with recreational pursuits. However, it is imperative to acknowledge that bariatric tourism is not devoid of its inherent challenges and associated risks [11]. These encompass issues such as the absence of stringent regulatory and accreditation standards, potential medical complications and infections, logistical intricacies concerning postoperative care and communication, and disparities in cultural and legal frameworks. Consequently, individuals contemplating bariatric surgery abroad must engage in comprehensive research and exercise discernment in selecting reputable and dependable service providers [12,13].

Recent empirical research underscores that a significant 2% of global bariatric surgeries cater to medical tourists. Countries such as Mexico, Lebanon, and Romania are prominent destinations, with a substantial influx of patients originating from the United States, the United Kingdom, and Germany. Predominant procedures include laparoscopic sleeve gastrectomy (LSG), laparoscopic Roux-en-Y gastric bypass (RYGB), and one anastomosis gastric bypass (OAGB) [14]. The motivation for patients opting for bariatric tourism often emanates from the scarcity of affordable domestic healthcare options and prolonged waiting periods.

Our center's two most commonly performed bariatric procedures were Roux-en-Y gastric bypass (60.2%) and sleeve gastrectomy (39.8%). These procedures align with international trends, as they are well-established methods for achieving substantial weight loss and managing obesity-related health issues [15].

This study further entails an in-depth analysis of data extracted from bariatric surgical centers catering to foreign patients. The findings underscore noteworthy deficiencies in accreditation standards, communication protocols, perioperative care, and the provision of travel health advice. To address these lacunae, we recommend the establishment of an international registry comprising accredited bariatric tourism providers, coupled with enhanced patient education initiatives.

Here we list a few destinations that have emerged as favored hubs for bariatric tourism:

Spain and Portugal offer cost-effective obesity treatments compared to other European alternatives, boasting a conducive climate and tourist-friendly culture.

Turkey has also been hailed as an attractive destination for bariatric tourism due to its geographic location, affordable living costs, and top-notch healthcare facilities.

India is driven by a high demand for bariatric procedures stemming from a substantial prevalence of obesity. It showcases a plethora of qualified surgeons offering diverse procedures at competitive prices and attracts patients from neighboring nations.

Due to their top-of-the-line healthcare facilities, the United States and the United Kingdom have always been attractive destinations for bariatric tourism.

Pakistan has recently emerged as an increasingly attractive destination for bariatric tourism. The country grapples with a substantial obesity prevalence, with over 30% of its adult population classified as overweight or obese [16]. This surge in obesity rates has driven a corresponding demand for bariatric surgery, accompanied by an influx of proficient and experienced surgeons offering these services. Pakistan's lower cost of living further enhances its appeal to international patients seeking cost-effective surgical options.

Our study looked into the motivations driving patients to choose Pakistan for bariatric surgery. The most prominent factor motivating patients was economic, with 33.3% citing cost savings as the primary driving force and 24.36% mentioning long waiting time as the second. This highlights the role of accessibility and timely care in the decision-making process for medical tourists.

Our center, Shifa International Hospital, Islamabad, Pakistan, is a high-volume center. During the past year, we performed 154 bariatric surgeries [17]. The average cost of a bariatric surgery in our center ranges from 2100-2300 USD. This is close to the surgical cost of around 2700 USD in Turkey and way less than the average cost of 7000 USD in India; both countries are attractive destinations for medical tourism. The cost in the United States is around 25000-27000 USD, and in the United Kingdom, it is about 13000-15000 USD, prices that are way more than our center is charging [18].

Patient satisfaction is of utmost importance regarding surgery and postoperative recovery. Results from our study showed a remarkably high satisfaction rate among the surveyed patients. An overwhelming majority (92.3%) expressed satisfaction with their surgical procedures. These results were consistent with multiple studies with varying satisfactory rates ranging from 85% to 91% [19,20]. This is a significant finding, as it suggests that bariatric surgery services provided in Pakistan are meeting or exceeding the expectations of international patients. High patient satisfaction is crucial in attracting medical tourists and sustaining a positive reputation in the medical tourism industry [13].

Waiting time is another factor influencing patients to seek out other avenues for bariatric surgery. Usually, at our center, there is no waitlist for patients who are ideal candidates for bariatric surgery. This starkly contrasts most of the first-world centers where waiting time stretches from 12 months to 5 years. A problem that was exacerbated with the COVID-pandemic [21,22].

The concept of surgical tourism in Pakistan, albeit in its infancy, is not entirely new in Pakistan. ALSA, a surgical tourism company based in Pakistan formed in 2008, has reported positive feedback from its patients, who have experienced significant weight loss and improved health outcomes following bariatric surgery. They have served patients from a diverse array of countries, including Canada, the United Kingdom, the United States of America, Saudi Arabia, the United Arab Emirates, Qatar, Bahrain, Oman, Kuwait, Iraq, Iran, Afghanistan, India, Bangladesh, Sri Lanka, and Nepal [18].

Similarly, in our study, a large group of patients came from Qatar, Afghanistan, the United Kingdom, the United Arab Emirates, Saudi Arabia, the United States of America, Canada, and Australia. This geographic diversity underscores the global appeal of Pakistan as a destination for bariatric surgery. The wide array of countries represented in the patient cohort suggests that Pakistan's medical tourism industry is catering to an international clientele.

This study provides valuable insights into the motivations and experiences of international patients seeking bariatric surgery in Pakistan. The exceptionally high level of patient satisfaction speaks to the quality of care provided by the medical institutions in Pakistan. However, further research could explore specific aspects of the patient experience, such as preoperative and postoperative care, to identify areas for improvement and maintain high levels of patient satisfaction.

## Conclusions

In conclusion, Pakistan can be as viable a destination for bariatric tourism when it comes to patient satisfaction as the countries mentioned above. The results show that our patients are not only satisfied with the surgery and surgical outcomes but also enjoy the advantages of shorter waiting times and economic viability. The data and analysis presented in this study shed light on the motivations and experiences of international patients traveling to Pakistan for bariatric surgery. These insights are invaluable for healthcare providers, policymakers, and the medical tourism industry as they seek to enhance the accessibility, affordability, and quality of healthcare services for domestic and international patients.

Additionally, future research could investigate the economic impact of bariatric surgery tourism on the local healthcare system and the broader economy, contributing to a comprehensive understanding of the implications of this growing phenomenon.

## Appendices

Questionnaire for bariatric tourism

Name

Age

Marital status

Address

1. Date of surgery

2. Which procedure did you underwent?

o   Roux-en-Y Gastric Bypass

o   Sleeve Gastrectomy

3. What motivated you to get the surgery done from Pakistan?

o   Financial Burden

o   Waiting period

o   Social support

o   Tourism

o   Advertisement

o   Privacy

o   Unavailability in home country

o   Better medical services

4. How Satisfied were you with your overall experience and weight loss?

o   Very Unsatisfied

o   Unsatisfied

o   No response

o   Satisfied

o   Very Satisfied

•       How satisfied were you with the surgical procedure?

o   Very Unsatisfied

o   Unsatisfied

o   No response

o   Satisfied

o   Very Satisfied

•       How satisfied were you with the hospital environment?

o   Very Unsatisfied

o   Unsatisfied

o   No response

o   Satisfied

o   Very Satisfied

•       How satisfied were you with staff?

o   Very Unsatisfied

o   Unsatisfied

o   No response

o   Satisfied

o   Very Satisfied

•       How satisfied were you with the financial burden?

o   Very Unsatisfied

o   Unsatisfied

o   No response

o   Satisfied

o   Very Satisfied

•       How satisfied were you with the waiting period?

o   Very Unsatisfied

o   Unsatisfied

o   No response

o   Satisfied

o   Very Satisfied

5. Any Post-op Complications you encountered?

6. How much weight did you lose?
